# PERSONA: A personalized model for code recommendation

**DOI:** 10.1371/journal.pone.0259834

**Published:** 2021-11-16

**Authors:** Tam The Nguyen, Tung Thanh Nguyen

**Affiliations:** Department of Computer Science and Software Engineering, Auburn University, Auburn, Alabama, United States of America; Vietnam National University, VIET NAM

## Abstract

Code recommendation is an important feature of modern software development tools to improve the productivity of programmers. The current advanced techniques in code recommendation mostly focus on the crowd-based approach. The basic idea is to collect a large pool of available source code, extract the common code patterns, and utilize the patterns for recommendations. However, programmers are different in multiple aspects including coding preferences, styles, levels of experience, and knowledge about libraries and frameworks. These differences lead to various usages of code elements. When the code of multiple programmers is combined and mined, such differences are disappeared, which could limit the accuracy of the code recommendation tool for a specific programmer. In the paper, we develop a code recommendation technique that focuses on the personal coding patterns of programmers. We propose Persona, a personalized code recommendation model. It learns personalized code patterns for each programmer based on their coding history, while also combines with project-specific and common code patterns. Persona supports recommending code elements including variable names, class names, methods, and parameters. The empirical evaluation suggests that our recommendation tool based on Persona is highly effective. It recommends the next identifier with top-1 accuracy of 60-65% and outperforms the baseline approaches.

## 1 Introduction

In software development, programmers must interact with different types of information and perform many activities to build an application. They constantly need to figure out which variables, objects, or methods to use next. Additionally, the number of objects and methods to use in the current project or libraries are often huge, which makes programmers impossible to remember all the usages. To help programmers work more effectively, modern integrated development environments (IDEs) offer code recommendation features. These tools help developers to complete the names of classes, methods, fields, and keywords. Murphy *et al*. [1] performed a study that indicates programmers could use the code recommender up to several times per minute when they develop applications in Eclipse.

However, the default code recommendation plugins inside current IDEs offer fairly limited functionalities. Firstly, the current recommendation tools often provide the ranking candidates based on alphabetical order. Certain candidates have a higher probability to appear than others, but might not be included at the top of the ranked list. The recommendation could be time-consuming if the number of candidates is big, and the user needs to move down the rank list to find what he wants. Secondly, the built-in tools often lack the consideration of code context when making a recommendation. For example, let us assume a user created a new URL object in the previous line, he is likely to create a HttpURLConnection object by calling the openConnection method on the newly created URL object. Thus, a tool should recognize the existence of the URL object as the context when making recommendations.

To further improve the effectiveness and usefulness of current code recommendation tools, multiple methods have been proposed [2–6]. Most of the techniques are motivated by the crowd-based approach. The approach focuses on the common code patterns of objects and methods that are shared among multiple programmers. The idea is to build a large dataset by collecting a large pool of available source code. Next, common code patterns are extracted or inferred from the dataset. In the recommendation phase, the current code context is used to match with learned code patterns to infer the recommendations.

At the same time, each programmer has certain coding preferences and styles. For example, a programmer could prefer to use CSVReader object to read a file, while others prefer to use BufferedReader. These coding preferences are referred to as personal coding patterns of programmers. In the crowd-based approach, while common code patterns are combined and inferred, such coding preferences are blurred. This could limit the accuracy of the code recommendation tool for a specific programmer. To capture the personal coding patterns, a code recommendation tool should take into consideration the code history of written the programmer. For example, which classes, objects, or code patterns that the programmer often uses. Providing such recommendations could improve the effectiveness and enhance the user satisfaction of the tool. Our preliminary study [7] shows a recommendation model that incorporates personal code patterns provides improvements in suggesting variable declaration and initialization code. Therefore, it is desirable to combine both personal and common code patterns to improve current code recommendation models.

In this paper, we propose Persona, a novel code recommendation model that focuses on the personal coding patterns of programmers while also combines with project-specific and common code patterns. As a personalized model, Persona is built and updated for each programmer. It is composed of three sub-models: PerCR, a model that captures personal code patterns of a programmer; ProCR, a model that captures the project-level code patterns that the programmer is working on; and GenCR, a general model that capture code patterns shared between multiple projects. Persona incorporates code patterns learned from the three sub-models together and utilizes those patterns for recommending code elements including variable names, class names, methods, and parameters.

Persona utilizes the fuzzy set theory [8] to model correlation/association between code elements. It defines a fuzzy set of potential recommendation candidates toward code elements that appear in the current code context. Each candidate has a membership score, which determines a certain degree of membership in the fuzzy set. The membership score is calculated based on various factors such as the code history of the programmer, the project he is working on, or common code patterns. The candidate with a higher membership score will be ranked higher in the recommendation list. The details of our approach are presented in Section 4.

To build the proposed recommendation model, we extract personalized object usage instances from the code history of a programmer. We use such data to train a personalized code recommendation model PerCR for the programmer. The code history of other programmers in the current project is also extracted to train a project-level recommendation model ProCR. We also train GenCR, a general model to capture common code patterns on a large code corpus. Finally, we incorporate the sub-models together to build Persona. Once trained, given the current editing code in which the programmer wants to invoke code recommendation, our recommendation tool extracts its context features and utilizes Persona to compute the recommendation rank list. The details of our recommendation system are presented in Section 5.

We have conducted several evaluation experiments to evaluate the usefulness and effectiveness of the personalized code recommendation approach. In the evaluation, Persona is trained on a big dataset, containing 14,807 Java projects across multiple domains, amounting to over 350 million lines of code in over 2 million files. Next, the model is evaluated on 10 large Java projects with the number of commits in each project is ranging from 23,000 to over 400,000. The evaluation results show that Persona could achieve high accuracy in code recommendation. For example, when evaluating Persona on a programmer, our approach has top-1 accuracy of 66% and top-3 accuracy of 74%. Furthermore, our model also outperforms the baselines significantly in top-1 accuracy in these experiments. It outperforms the first baseline by an average of 12-15%, and generates a gap of 4-6% when compared to the second baseline. We also show that the recommendation accuracy of Persona improves over time as more code of the programmer is used to train. By incorporating three sub-models together, the Persona performs reasonably well even if the code history of the programmers is thin in the project. The details of our evaluation process are presented in Section 6.

The key contributions of our paper include:

- We proposed Persona, a lightweight code recommendation model that focuses on the personal coding patterns of programmers. Persona is built and updated for each programmer. To learn personal coding patterns, it utilizes fuzzy logic to model correlation/association between code elements in the code history written by the programmer. Persona also incorporates project-specific and common code patterns efficiently to further improve the recommendation accuracy.
- We implemented a robust code recommendation system based on Persona. The system includes a module to extract the usages of variables, methods, classes, parameters from the code history of a programmer, as well as from a large codebase. The system is designed to train Persona efficiently. Furthermore, it also allows Persona to be re-trained easily to update the coding preferences of programmers as more training data becomes available.
- We performed an extensive evaluation that shows the effectiveness of the approach in code recommendation. Persona is trained on a dataset containing 14,807 Java projects, with over 350 million lines. We evaluated the model on 10 big Java projects with the number of commits in each project is ranging from 23,000 to over 400,000. The evaluation results show that Persona could achieve high accuracy in code recommendation and outperforms the baselines significantly. We also showed that the model could be re-trained and improves the recommendation accuracy over time as more code of the programmer is available for training.

The rest of the paper is organized as follows. The related work is presented in Section 2. In Section 3, we present the motivation of our approach. Next, we describe our proposed model in Section 4. The description of our code recommendation system is presented in Section 5. We present our evaluation in Section 6. Section 7 presents our discussion. Finally, we conclude the paper in Section 8.

## 2 Related work

There are various code recommendation techniques have been proposed over years, including [2–5, 9–11]. Bruch *et al*. [2] proposed three example based code completion systems where examples are extracted automatically from the example code base. SLANG [4] uses statistical language models such as *n*-grams, and RNNs to model application programming interface (API) method usages and recommend the next API method call. Grapacc [5] is a graph-based, pattern-oriented, context-sensitive code completion approach that models API usage patterns as frequent graph-based models. DroidAssist [3, 9] models the usage of API objects and methods based on Hidden Markov Models and provides recommendation on method calls. Precise [10] builds a parameter usage database based on the existing code base and recommends API parameters. Graphite [11] is an active code completion architecture that allows library developers to introduce interactive and highly-specialized code generation interfaces directly into the editor. Most of the current code recommendation techniques focus on modeling common code patterns from a large code base, then utilize the patterns to make recommendations.

The statistical approach for capturing rules and patterns in source code has become a hot research topic in software engineering in recent years. Hassan *et al*. [12] indicated “natural” software analytics based on statistical modeling will become one of the most important aspects of software analytics. Hindle *et al*. [13] shows that source code is repetitive and predictablelike natural language and they adopted an *n*-gram model on lexical tokens to suggest the next token. SLAMC [14] represents code by semantic tokens, i.e. annotations of data types, method/field signatures, etc. rather than lexical tokens. SLAMC combines *n*-gram modeling of consecutive semantic tokens, topic modeling of the whole code corpus, and bi-gram of related API functions. Tu *et al*. [15] exploited the localness of source code. White *et al*. [16] proposed deep learning approach modeling source code. Allamanis and Sutton [17] train an *n*-gram language model on a giga-token source code corpus. NATURALIZE [18] uses an *n*-gram language model to learns the style of a codebase and suggest natural identifier names and formatting conventions. Jacob *et al*. [19] uses an *n*-gram model to learn code templates. Hidden Markov Model has been used to infer the next token from user-provided abbreviations [20] and detect coded information islands, such as source code, stack traces, and patches, from free text [21]. Maddison *et al*. [22] proposed tree-based generative models for source code. Hsiao *et al*. [23] learns an *n*-gram language model on program dependence graph and uses the model for finding plagiarized code pairs. Nguyen *et al*. [24] introduced GraLan, a graph-based statistical language model that learns common API usage (sub)graphs from source code.

Pattern mining approaches represent usage patterns using various data structures such as sequences, sets, trees, and graphs. JADET [25] extracted a usage model as a set of partial order pairs of method calls. MAPO [6] mined frequent API call sequences and suggests associated code examples. Wang *et al*. [26] mines succinct and high-coverage API usage patterns from source code. Acharya *et al*. [27] proposed an approach to mine partial orders among APIs. Buse and Weimer [28] propose an automatic technique for mining synthesizing succinct and representative human-readable API examples. Other techniques include mining associate rules [29], item sets [2], subgraphs [30, 31], code idioms [32], etc.

Several approaches have been proposed to improve code recommendation/completion tasks. Robbes *et al*. [33] gathered information to improve code recommendation by replaying the entire change history of programs with the completion engine. In [34], the authors proposed and implemented new strategies for sorting, filtering, and grouping APIs in the code recommendation popup pane to improve accuracy. Hill and Rideout [35] proposed a method to match the code fragment under editing with small similar-structure code segments that often appear in large software projects. The authors of [36, 37] proposed methods to suggest source code examples to developers based on API documentation. Holmes and Murphy [38] described an approach to recommend code examples based based on heuristically matching with the structure of the code.

In general, personalized models have been studied extensively in the fields of recommender systems [39, 40] and collaborative filtering [41, 42]. For example, Hwang *et al*. [40] proposed a new recommender system, which employs a genetic algorithm to learn personal preferences of customers and provide tailored suggestions. In software engineering, several personalized approached has been proposed. Jiang *et al*. [43] developed a separate prediction model for each developer to predict software defects. In [44], the author proposed a personalized defect prediction framework that gives instant feedback to the developer at change level, based on historical defect and change data. Wang *et al*. [45] proposed a context-aware personalized task recommendation approach to aid dynamic worker decision in selecting crowd-testing tasks.

Fuzzy-based approaches have been proposed to solve problems in software engineering, such as bug triaging problem [46, 47], automatic tagging [48], bug categorization [49]. However, they focus on modeling textual software artifacts.

## 3 Motivation

**Fig 1:**
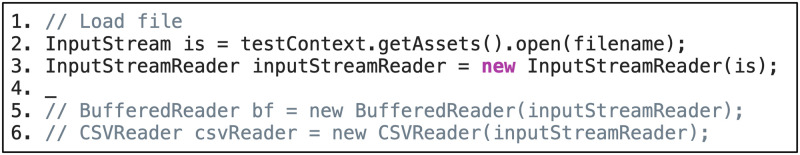
A code recommendation scenario.

Let us start with an example that explains the challenges when using the current code recommendation methods, and motivates our approach. Fig 1 shows a code recommendation scenario in which the programmer writes code to read a file. In the first line, he creates an InputStream object from the filename. Next, he creates an InputStreamReader object from the InputStream. Let us assume that he invokes code recommendation at the first of line 4. A code recommendation method based on the crowd-based approach would recommend creating a BufferedReader (line 5). This is because using a BufferedReader to read a file from an InputStreamReader is a common code pattern that is often shared between programmers. The model learns the pattern from mining a code corpus.

The preference of the programmer in the example is different. He prefers to use CSVReader object to read file instead of BufferedReader. He has been using CSVReader throughout his application development. Thus, an ideal code recommendation tool should prioritize the personal code patterns and recommends CSVReader (line 6).

The example shows that programmers have preferences and styles when coding including naming variables, using certain classes, objects, and methods, or applying certain coding patterns. Thus, such personal preferences should be taken into consideration when providing code recommendations as it could improve the effectiveness and enhance user satisfaction of the recommendation tool.

## 4 Model

In Persona, code recommendation is modeled as a ranking problem: given the current editing code *E* in which a programmer is asking recommendations for a missing code element, Φ is the set of all possible recommendation candidates, find a candidate *c* ∈ Φ with the highest possibility to be filled in the current missing location.

The key idea of Persona is to rank potential candidates *c* toward a set of context features *F* in *E* by modeling the correlation/association of *c* with each context feature in *F*. The set of feature *F* includes object types, method calls, variable names, and parameters that occur in *E*. If a candidate *c* has a higher correlation with features in *F*, *c* is considered to have a higher possibility and will be rank higher in the list.

For example, in Fig 1, in which the programmer invokes code recommendation at beginning of line 4. The goal of Persona is to rank CSVReader as the declaration type with highest possibility. The set of context features *F* includes object types {InputStream, InputStreamReader}, variables {is, testContext, inputStreamReader}, method calls {getAssets, open, InputStreamReader.new }, and parameters {fileName}.

To model correlation/association between candidates and context features, Persona utilizes the fuzzy set theory [8]. It defines a fuzzy set of potential candidates toward a context feature as follows.

**Definition 1 (Potential candidate)** *For a specific context feature f, a fuzzy set C_f_, with an associated membership function μ_f_*(), *represents the set of potential candidates toward f, i.e. candidates that are highly correlated with f*

Fuzzy set *C*_*f*_ is determined via a membership function *μ*_*f*_() with values in the range [0, 1]. For a candidate *c*, the membership score *μ*_*f*_(*c*) determines the certainty degree of the membership of *c* in *C*_*f*_, i.e. how likely does *c* belong to the fuzzy set *C*_*f*_. *μ*_*f*_(*c*) represents the degree of association between *c* and *f*. *μ*_*f*_(*c*) also determines the ranking of *c* toward *f*. If *μ*_*f*_(*c*)>*μ*_*f*_(*c*′) then *c* is considered higher correlated to *f* to *c*′. The membership score is often computed as follows.

**Definition 2 (Membership score)** *The membership score μ_f_(c) is computed as the correlation between the set D_f_ representing usages of the context feature f, and the set D_c_ representing usages of the candidate c*:

$$\begin{array}{c} \mu_{f}\left(c\right)=\frac{|D_{f}\cap D_{c}|}{|D_{f}\cup D_{c}|}=\frac{n_{f,c}}{n_{f}+n_{c}-n_{f,c}} \end{array}$$ (1)

where, *n*_*f*_ is the number of usages of the context feature *f*, *n*_*c*_ is the number of usages of the candidate *c*, and *n*_*f*,*c*_ is the number of times that the candidate *c* co-occurs with *f*. As the Eq 1, the value of *μ*_*f*_(*c*) is between [0, 1]. If *μ*_*f*_(*c*) = 1, then *c* always occurs on the code snippets that contain *f*, thus, given a code snippet contains *f*, it is very likely that *c* co-occurs. If *μ*_*f*_(*c*) = 0, it means that *c* never occurs on code snippets that contains *f*, thus, given a code snippet contains *f*, it is unlikely to recommend *c*. In general, the more frequently *c* co-occurs with *f*, the higher value of *μ*_*f*_(*c*).

Based on the fuzzy logic framework described above, we develop three different code recommendation models. Each model has its membership score function (Eq 1) and is learned from different datasets. Finally, we incorporate the three models together to build Persona. Let us describe each model as follows.

### 4.1 Personalized code recommendation model

As demonstrated in Section 3, programmers have different coding preferences, styles, experience levels, and knowledge about libraries and frameworks. For example, a programmer might prefer using certain classes or methods than others; some programmers prefer short variable names for a BufferedReader object such as b or bf, while others use long names such as bufferedReader, etc. In other words, there are personal code patterns that appear in the code written by a programmer. Thus, a code recommendation model that utilizes those personal code patterns could improve the code recommendation performance significantly. Based on this observation, we design a personalized fuzzy-based code recommendation model (or PerCR for short).

Let us assume a programmer *d* is working on a project *P*, *H*_*d*_ is the code history written by the programmer in the current project. The membership score in PerCR is defined as:

$$\begin{array}{c} \mu_{d}\left(f,c,H_{d}\right)=\frac{n_{f,c}\left(H_{d}\right)}{n_{f}\left(H_{d}\right)+n_{c}\left(H_{d}\right)} \end{array}$$ (2)

where *μ*_*d*_(*f*, *c*, *H*_*d*_) represents the membership score of candidate *c* in the fuzzy set *C*_*f*_ of the context feature *f*, *n*_*f*_(*H*_*d*_) represents the usages of *f* in *H*_*d*_, *n*_*c*_(*H*_*d*_) represents the usages of *c* in *H*_*d*_, and *n*_*f*,*c*_(*H*_*d*_) represents the usages in *H*_*d*_ which *f* and *c* co-occur. Normally, *n*_*f*_(*H*_*d*_) is defined as the number of occurrences of *f* in *H*_*d*_. However, in PerCR we also want to model the change in code patterns of programmers over time. For example, a programmer might start by using the BufferedReader object to read file but as he writes more code, he gradually changes his preference to using CSVReader. Thus, in PerCR, *n*_*f*_(*H*_*d*_) is computed as follows:

$$\begin{array}{c} n_{f}\left(H_{d}\right)=\sum_{x\in H_{d}:f}e^{-\frac{1}{\Delta t_{x}}} \end{array}$$

where Δ*t*_*x*_ = *t*_*x*_ − *t*_0_ is the time decay, *t*_0_ is the timestamp in which the project start, *t*_*x*_ is the timestamp in which *f* occurs in *H*_*d*_. The idea behind the formula is that the occurrence of *f* later in the project has more influence over the previous occurrences. Similarly, we have:

$$\begin{array}{c} n_{c}\left(H_{d}\right)=\sum_{x\in H_{d}:c}e^{-\frac{1}{\Delta t_{x}}}\;\text{and}\;n_{f,c}\left(H_{d}\right)=\sum_{x\in H_{d}:f,c}e^{-\frac{1}{\Delta t_{x}}} \end{array}$$

### 4.2 Project-level code recommendation model

When multiple programmers work on the same project, they read, share, and reuse the code of each other. Thus, the code written by a programmer could be influenced by other programmers in the same project. For example, a programmer could create and use MapUtil class that contains several utility methods for Map. Other programmers in the same project also reuse the class. Thus, the code patterns related to the class could be shared between programmers in the project. We present ProCR, a fuzzy-based model that captures the project-level code patterns in the project that the programmer is working on.

Let us assume a programmer *d* is working on a project *P*, and *P* − *H*_*d*_ is the code history written by all other programmers (except *d*) in the project. ProCR is the project-level code recommendation model defined specifically for the programmer *d*. The membership score in ProCR is defined as:

$$\begin{array}{c} \mu_{d}\left(f,c,P-H_{d}\right)=\frac{n_{f,c}\left(P-H_{d}\right)}{n_{f}\left(P-H_{d}\right)+n_{c}\left(P-H_{d}\right)} \end{array}$$ (3)

where *μ*_*d*_(*f*, *c*, *P* − *H*_*d*_) represents the membership score of candidate *c* in the fuzzy set *C*_*f*_ of the context feature *f*. Other terms in Eq 3 are defined similarly to corresponded terms in Eq 2. In other words, the project-level model ProCR is defined similarly to the personalized model PerCR. The difference is that PerCR is trained from the code history *H*_*d*_ of the programmer *d*, while ProCR is trained on the code history of other programmers in the same project.

### 4.3 General code recommendation model

In modern application development, programmers rely heavily on shared APIs to write code. For example, two different programmers could use the same API classes such as BufferedReader, File to read data from a file. The usage pattern of using those objects could be similar between the two programmers. Programmers might also share programming conventions of programming languages such as naming conventions. Thus, programmers do share common code patterns and we want to incorporate these patterns to our approach to improve the recommendation accuracy. We propose GenCR, a fuzzy-based model that captures such common code patterns shared between multiple projects. The membership score of GenCR is defined as follows:

$$\begin{array}{c} \mu \left(f,c,\bar{P}\right)=\frac{n_{f,c}\left(\bar{P}\right)}{n_{f}\left(\bar{P}\right)+n_{c}\left(\bar{P}\right)-n_{f,c}\left(\bar{P}\right)} \end{array}$$ (4)

where $\bar{P}$ is the set contains the code of all projects in the dataset except the current project *P*, $n_{f}\left(\bar{P}\right)$ is the number of occurrences of *f* in $\bar{P}$, $n_{c}\left(\bar{P}\right)$ is the number of occurrences of *c* in $\bar{P}$, and $n_{f,c}\left(\bar{P}\right)$ is the number of times that the candidate *c* co-occurs with *f* in $\bar{P}$.

### 4.4 Combining sub-models

Using each sub-model described above separately could yield a low-accuracy recommendation. For example, if a programmer just joined the project or the project just started, there is not much data to train PerCR and ProCR. Thus, these models could be fairly inaccurate, while GenCR could not recommend personal or project-level code patterns. To maximize the recommendation accuracy, we design Persona to incorporate the three sub-models together. It defines the membership score *μ*_*f*_(*c*) in Persona is as follows:

$$\begin{array}{c} \mu_{d}\left(f,c\right)=\alpha_{1}\mu_{d}\left(f,c,H_{d}\right)+\alpha_{2}\mu_{d}\left(f,c,P-H_{d}\right)+\alpha_{3}\mu \left(f,c,\bar{P}\right) \end{array}$$ (5)

where *α*_1_ + *α*_2_ + *α*_3_ = 1 are weighting coefficients. The value of *α*_*i*_ represents the contribution level of a sub-model towards Persona, the higher value of *α*_*i*_ the bigger contribution of the sub-model. If the model defines the membership score *μ*_*f*_(*c*) using Eq 5, we call the model PersonaSum.

As the sub-models are defined in separated datasets, the membership score of Persona could also be defined using the max function:

$$\begin{array}{c} \mu_{d}\left(f,c\right)=\text{max}\left(\mu_{d}\left(f,c,H_{d}\right),\mu_{d}\left(f,c,P-H_{d}\right),\mu \left(f,c,\bar{P}\right)\right) \end{array}$$ (6)

In Eq 6, the sub-model with the highest value of membership score will decide the value of *μ*_*d*_(*f*, *c*). If the model defines the membership score using Eq 6, we call the model PersonaMax. We experimented both approaches of calculating *μ*_*d*_(*f*, *c*) in Persona in our evaluation.

After defining the membership score function, we show how Persona calculates the rank list of candidates using the fuzzy set theory. Based on the definition of potential candidates toward a context feature *f* as a fuzzy set (Definition 1), Persona defines potential candidates toward a set of context features *F* using the union operation of fuzzy set theory as follows.

**Definition 3** *Given a set of context features F, a fuzzy set C_F_, with an associated membership function μ_F_*(), *represents the set of potential candidates toward F, i.e. the candidates that are highly correlated with context features of F. C_F_ is computed as the union of the fuzzy sets C_f_ of context features in F*:

$$\begin{array}{c} C_{F}=\underset{f\in F}{\cup }C_{f} \end{array}$$ (7)

Because *C*_*F*_ is a fuzzy set, it has a membership function *μ*_*F*_. The union operation in fuzzy logic is defined via calculating *μ*_*F*_ from $\mu_{f_{1}}...\mu_{f_{k}}$. There are several equations for fuzzy union operation, we use the following one:

**Definition 4** *The membership score μ_F_(c) is calculated as the combination of the membership scores μ_f_(c) of its associated context feature f*:

$$\begin{array}{c} \mu_{F}\left(c\right)=1-\prod_{f\in F}\left(1-\mu_{f}\left(c\right)\right) \end{array}$$ (8)

In Eq 8, *μ*_*F*_(*c*) represents the correlation of candidate *c* toward a set of context features *F*. As the equation, we see that the value of *μ*_*F*_(*c*) is also between [0, 1] and represents the likelihood in which the candidate *c* belongs to the fuzzy set *C*_*F*_, i.e. the set of potential candidates for the set of context features *F*. *μ*_*F*_(*c*) = 0 when all *μ*_*f*_(*c*) = 0, which means that *c* never occurs on any code contains a context feature in *F*. Thus, Persona considers that *c* is unlikely to occur on the code contains *F*. If there is any method *f* is *F* with *μ*_*f*_(*c*) = 1, then *μ*_*F*_(*c*) = 1, or Persona considers that *c* is very likely to occur on the code contains *F* as *c* always occurs on code contains *f* in *F*. In general, the more context features *f* in *F* with high *μ*_*f*_(*c*) values, the higher *μ*_*F*_(*c*) is, or *c* is more likely to occur on the code contains *F*.

In the code recommendation phase, Persona ranks candidates based on the value of *μ*_*F*_(*c*) and provides the rank list for the user. The higher value of *μ*_*F*_(*c*), the higher ranking of the candidate *c* in the list.

## 5 System implementation

### 5.1 Overview

**Fig 2:**
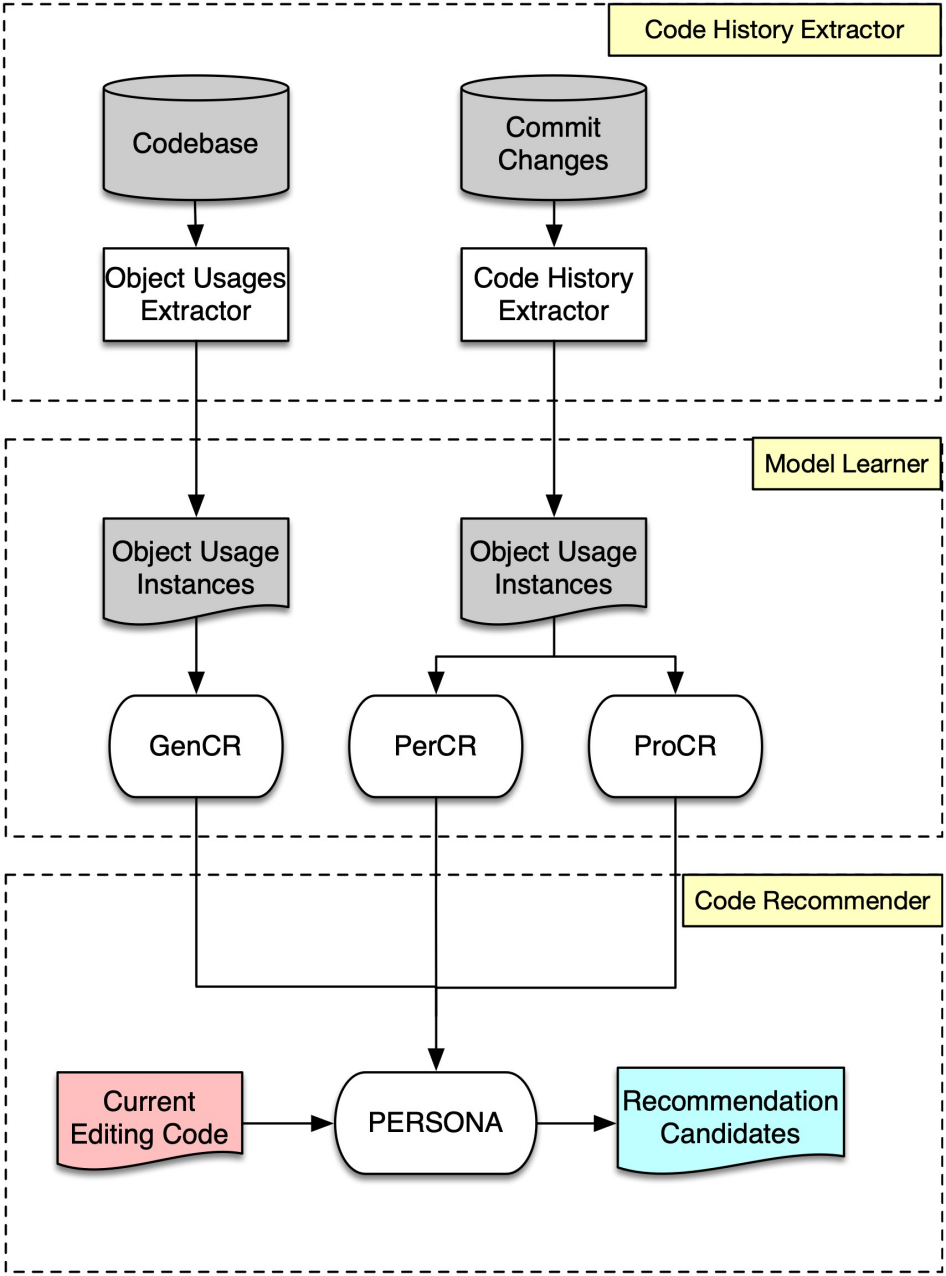
Overview of the system.

In this section, we briefly discuss the points in the design and implementation of our recommendation system. Fig 2 shows an overview of the system. Overall, it consists of 3 main components. The code history extractor is the component for extracting personalized object usage instances from the code history of a programmer. The model learner uses the extracted data to train and incorporate the three sub-models in Persona. Finally, the code recommender utilizes the personalized model to make recommendations on the current editing code.

### 5.2 Code history extractor

**Fig 3:**
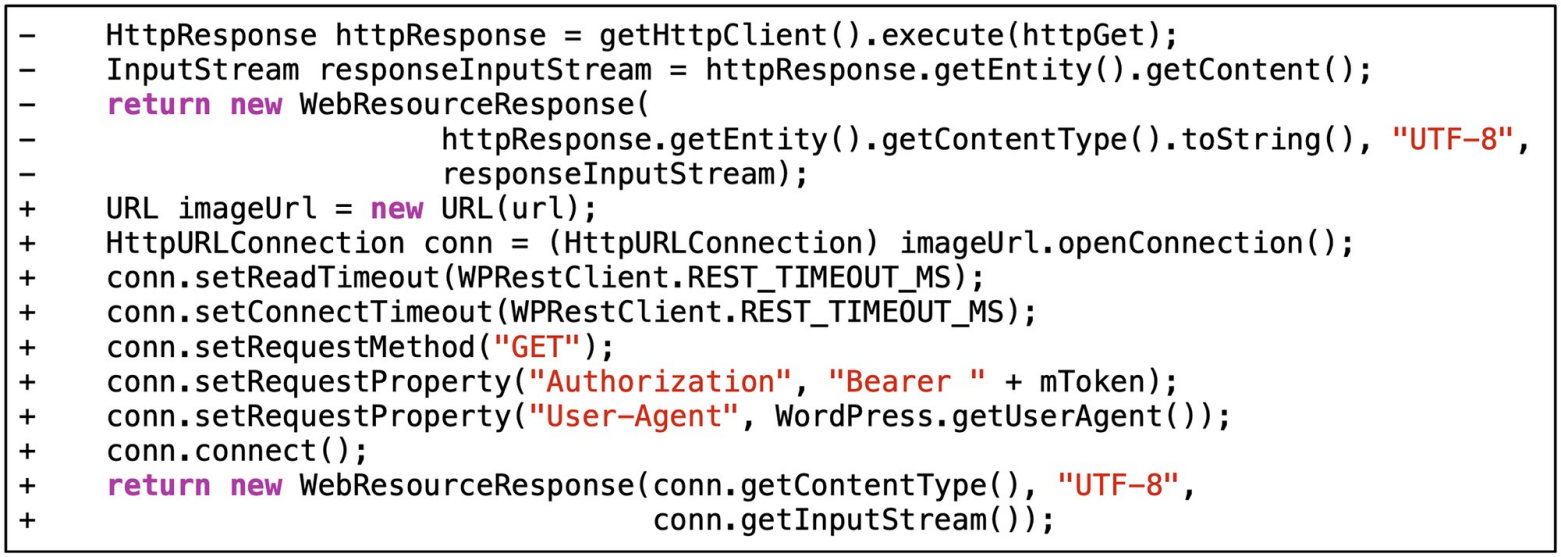
An example of code changes in a commit.

Because our recommendation techniques are learned from the personalized code history of programmers, we have built a code history extractor module for extracting usages of variables, methods, classes, and parameters of a programmer from his code development history. Typically, whenever a programmer adds a new code or updates the current existing code, he will submit a commit to the version control system. Fig 3 shows an example of code changes in a commit of a programmer. In the example, the programmer switched from using a HTTPResponse object to a HttpUrlConnection object. In our approach, we extract personalized code patterns from code changes in commits. In particular, for each code change in a commit of the programmer, the code history extractor will analyze the post-commit version, and extract object usages in the new code that the programmer added.

To extract the usages of variables, methods, classes, and parameters, the extractor uses Groum (Graph-based Object Usage Model) [30] to represent the object usages in the source code. Groum is a graph that represents the object usages in source code. It has two kinds of nodes: *object nodes* and *action nodes*. An object node represents an object. It is labeled by the name of the object type (e.g. HttpURLConnection). An action node represents a method call. It is labeled the method qualified name (e.g. URL.openConnection). There are two kinds of edges representing control flow between action nodes and data flow between action nodes and object nodes.

In Groum, each object created or involved during the execution is represented as an object node. We also treat primitive variables as object nodes. Action nodes represent any action that is performed on object nodes. Action nodes could be object instantiations, method calls, data field accesses of one object, or other operations. Object nodes are labeled by class names (object nodes represent primitive variables are labeled by types). Action nodes of types object instantiations, method calls, or data field accesses are labeled as “C.m” where C is its class name and m is the method (or field) name. Other action nodes that represent operations are labeled as the name of the operation.

The control edges of Groum are used to represent the temporal orders between action nodes. A control edge from an action node A to action node B means that A is executed before B in the execution path. Because Groum is defined for each execution path, thus, there is only one temporal order between action nodes, which is represented by a set of control edges between action nodes. The data edges indicate the data dependencies between data nodes and action nodes. A data edge from object node A to action node B means that A is a parameter of the action that B represents. A data edge from an action node B to data node A means the action node B returns the object node A.

**Fig 4:**
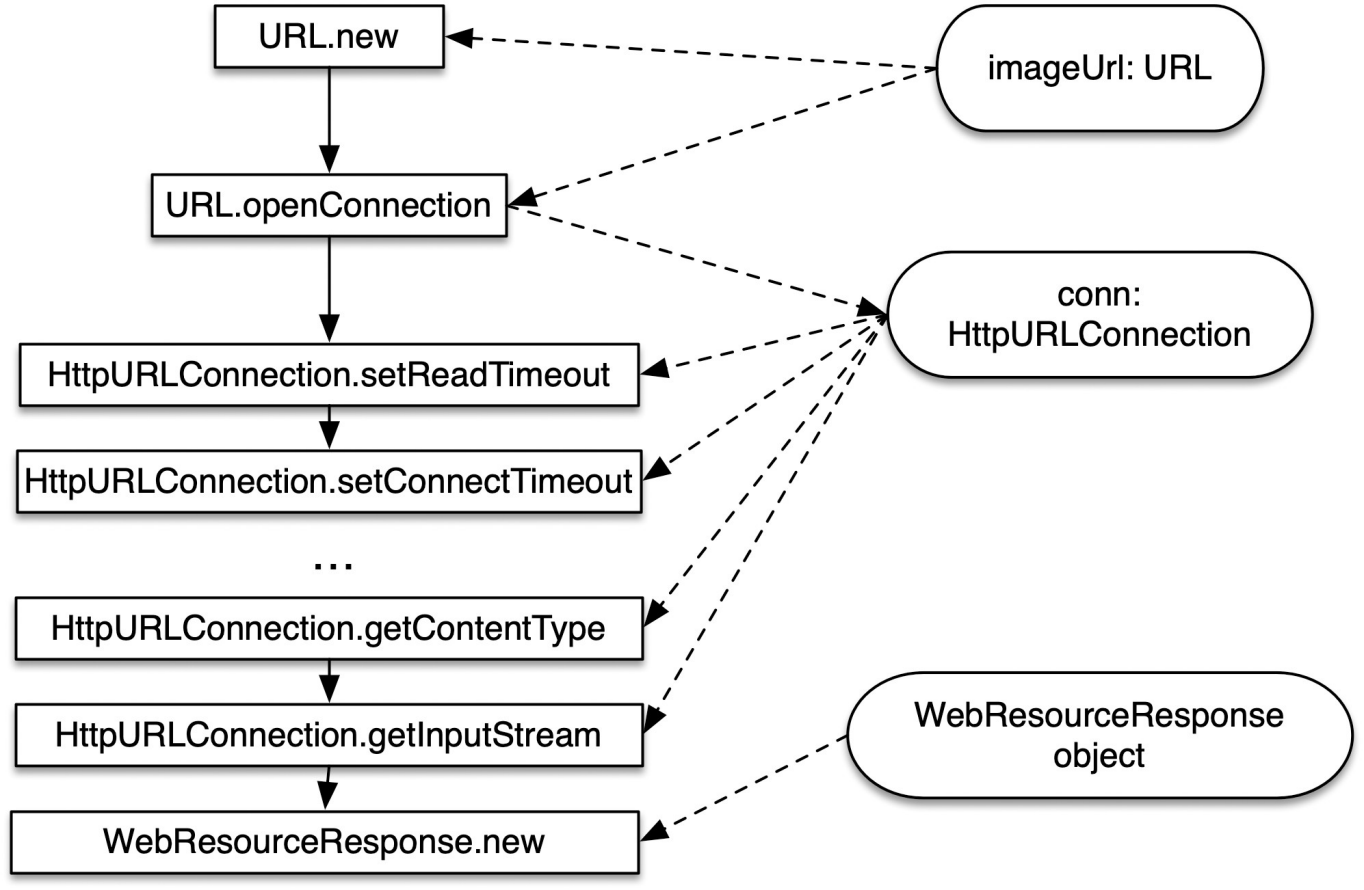
The extracted Groum of the added code.

Fig 4 illustrates the Groum of the code that the programmer added in the commit. Rectangle nodes are action nodes, while object nodes are represented as round rectangle nodes. Solid arrows represent the control edge between action nodes and dashed arrows represent data edges. The algorithms are used to extract Groum from source code could be found at [3, 9, 30].

There are several advantages of using Groum to represent and extract the usages of variables, methods, classes, and parameters. Firstly, it removes redundant information in code such as keywords (try, return,…), or symbols (=,+…), and only focuses on important information such as objects, method calls. Secondly, Groum avoids the problem of duplicated counting when extracting the occurrences of code elements and co-occurrences between code elements. For example, in Fig 4, the variable name conn appears multiple times in the code. Using Groum, all the appearances are traced back to a single object node. Thus, the occurrence of conn is counted as one and the co-occurrence between conn and other code elements is also counted as one.

To produce the training data for Persona, the extractor travels through nodes in Groum and counts the occurrences and co-occurrences between code elements. Note that, for each occurrence of a code element, we also store the timestamp in which the programmer added it to the project. The time information is important when training the personalized model. To train the sub-model GenCR, we also developed a code extractor to extract Groum from source files in a code corpus.

### 5.3 Learning recommendation models

We train each sub-model in Persona separately. To train the personalized model PerCR, we need to calculate *n*_*f*,*c*_(*H*_*d*_), *n*_*f*_(*H*_*d*_), *n*_*c*_(*H*_*d*_) in Eq 2. Calculating these values requires counting the occurrences of code elements and co-occurrences between code elements in the code history of the programmer. We explained the counting process in the previous section. Training the project-level model ProCR is similar to PerCR, the only difference is that ProCR is trained on the code history of other programmers in the current project. To train GenCR, we collect a code dataset contains multiple projects. Next, we obtain the source files from the projects and extract Groum from source files. GenCR is trained by computing values $n_{f,c}\left(\bar{P}\right),n_{f}\left(\bar{P}\right),n_{c}\left(\bar{P}\right)$ described in Eq 4. Finally, we incorporate the three sub-models with either Eqs 5 or 6.

### 5.4 Recommending code

Let us go back to the scenario in Fig 1. The programmer writes code to read a file. In the first line, he creates an InputStream object from the filename. Next, he creates an InputStreamReader object from the InputStream. Let us assume, he invokes code recommendation at the first of line 4. Upon the request, our tool will analyze the current edding code, build a temporary Groum, and extract the set of context features *F* includes object types {InputStream, InputStreamReader}, variables {is, testContext, inputStreamReader}, method calls {getAssets, open, InputStreamReader.new }, and parameters {fileName}.

In the next step, the tool will build a set of candidates for recommendation. It starts by analyzing which types of code elements are asked for the recommendation. In the example, the candidates should be a class or a variable. All classes and variables that are available in the current editing code will be added to the set of candidates. The tool then utilizes Persona to calculate the relevance score of each recommendation candidate towards the set of context features *F* using Eq 8. The set of candidates will be sorted by the relevance score. Finally, the recommendation tool returns the rank list of candidates with relevant scores for the programmer to consider. Note that, if the user requests recommendations for a new variable name, the tool will consider all the variable names has been used for the object before as the candidates. These names are stored in our model and might not be in the editing code.

## 6 Evaluation

We conducted several experiments to evaluate the effectiveness of our approach to learning and recommending code for programmers. All experiments are executed on a computer running Windows 10 with Intel Core i7 3.6Ghz CPU, 16GB RAM, and 1TB HDD storage. To conduct the evaluation, we collected a dataset consists of multiple Java projects that have source code repositories available on GitHub. The dataset that we used was carefully collected and studied by Allamanis *et al*. [17]. The corpus can be found and downloaded online [50]. It contains 14,807 projects across a wide variety of domains amounting to over 350 million lines of code in over 2 million files. The number of code tokens in the dataset exceeds 1.5 billion. Note that the dataset only contains the .java extension files, it does not contains revisions or commit changes. The characteristics of the corpus are shown in Table 1. We call this dataset A14K.

**Table 1:** Dataset characteristics.

Number of projects	14,807
Number of files	2,130,264
Number of lines of code	352,312,696
Number of code tokens	1,501,614,836

Because the A14K dataset only contains a snapshot of .java files, it is only used for training GenCR and the baselines. For evaluating Persona, it is required to have the code history of projects and programmers. Thus, we manually selected 10 projects in the dataset to evaluate our model. We selected such projects by first sorting the projects in the dataset by the number of commits. Next, we chose projects that have the highest number of commits while the vast majority of code is written in Java. We avoided selecting certain projects. First, we avoided projects that share duplicated code with a previously selected project. We also avoided projects are developed in multiple programming languages. For each selected project, we checked out its source code repository to retrieve all the code and commit changes. Table 2 shows the list of selected projects along with the number of contributors and commits.

**Table 2:** Projects used in the evaluation.

Name	Description	#Contributors	#Commits
liferay-portal	Enterprise web platform	553	407,015
intellij-comm	IDE for Java	510	279,093
osmand	Android map app	688	62,493
geogebra	Multi-platform math app	24	52,750
elasticsearch	Search engine	1,415	52,628
camel	Middleware framework	609	45,158
lucene-solr	Enterprise-search platform	181	33,654
cdt	IDE for C++	266	34,603
cassandra	Database system	298	25,289
hadoop	Data processing framework	278	23,877

### 6.1 Settings and baselines

For each selected project, the set of commits is sorted in chronological order. Next, we grouped commits by programmers. When we perform an evaluation experiment for a programmer *d*, his commit set is divided into a training set *TR*_*d*_ and testing set *TE*_*d*_ chronologically. The training set *TR*_*d*_ is used to train the sub-model PerCR. The code in the testing set *TE*_*d*_ is used to evaluate later. In our experiments, the training set is selected as the first 70% of the initial set of commits in chronological order, while the remaining 30% of the commits are used as the testing set. The sub-model ProCR is trained on the set of commits of other programmers. The commits that are used to train PerCR appear before the first commit of the testing set *TE*_*d*_. The sub-model GenCR is trained on the initial dataset, which contains a snapshot of .java files (the current project is excluded). Finally, we combine the sub-models using both Eqs 5 and 6. In the first method, we set the weighting coefficients equally, $\alpha_{1}=\alpha_{2}=\alpha_{3}=\frac{1}{3}$. The second method uses the max function to combine sub-models. The two approaches are called PersonaSum, and PersonaMax correspondingly.

In our evaluation, to compare our model with the baselines, we chose the task of recommending the next identifier in a code sequence. The types of identifiers that we considered include variable and field names, type names such as class and interface names, method names, and parameters. Given a code sequence, a recommendation model is expected to recommend the most probable identifier. Alamalis *et al*. [17] shows that learning to predict code elements is difficult mainly because of the identifiers. Thus, we chose this task to better compare the effectiveness of recommendation models. This evaluation task has been used similarly in the evaluation of prior approaches [13–15].

Recommendation accuracy is measured as follows. Our evaluation tool predicts and evaluates *all identifiers* in every code sequence from the testing set. At a position *i*, it uses the recommendation model under evaluation to compute the top *k* most likely identifiers *x*_1_, *x*_2_, …, *x*_*k*_ for that position based on the previous code tokens. If the actual identifier *s*_*i*_ at position *i* is among *k* suggested results, we count this as a hit. The top-*k* suggestion accuracy for a sequence is the ratio of the total hits over the sequence’s length. For example, if we have 70 hits on a code sequence of lengths 100 for a test file, accuracy is 70%. The top-*k* accuracy is the ratio of the total hits over the total number of evaluated tokens.

To compare the effectiveness of Persona, we chose two baseline models: *n*-gram and recurrent neural network (RNN) for comparison due to the following reasons. First, both of them are popular statistical models to capture common patterns in a large dataset and are comparable with Persona. In addition, *n*-gram is widely used in recent research on code recommendation [13–15]. Raychev *et al*. [4] and White *et al*. [16] recently evaluated RNN and *n*-gram in code recommendation, and reported RNN as the better approach. Note that our model and the baselines use the same 14K dataset as the training set.

An ***n*-gram model** is a simple statistical model for modeling sequences. An *n*-gram model learns all possible conditional probabilities *P*(*m*_*i*_|*m*_*i*−*n*+1_…*m*_*i*−1_), where *m*_*i*_ is the current code token and *m*_*i*−*n*+1_…*m*_*i*−1_ is the sub-sequence of *n* − 1 prior tokens. This is the probability that *m*_*i*_ occurs as the next code tokens of *m*_*i*−*n*+1_…*m*_*i*−1_. Using the chaining rule, we can use an *n*-gram model to compute the generating probability of any given sequence *m*_1_…*m*_*n*_. To improvement the effectiveness of *n*-gram model, Tu *et al*. [15] introduced CacheLM, a novel cache language model that consists of both an *n*-gram and an added “cache” component to exploit localness. The cache is the set of code tokens that appear in the same project as the test file. We re-implement this method for comparison. We used the same settings with the original model, i.e. 3-gram with “5K tokens” cache size. The model is trained on the A14K dataset with the cache is extracted from the project under test.

A **Recurrent neural network** (RNN) is a class of neural networks for learning sequences. A single-layer RNN can be trained with a collection of code token sequences and can compute the probability of the next code token for any given sequence. In other words, the RNN can compute all conditional probabilities *P*(*m*_*i*_|*m*_1_…*m*_*i*−1_) for any given sequence *m*_1_…*m*_*n*_. To do that, it maintains a context vector (hidden state) *c*_*i*_ represents current context of sub-sequence up to *m*_1_…*m*_*i*−1_. A function *f* is learned from data to compute the context vector at position *i*, *c*_*i*_ = *f*(*m*_*i*_, *c*_*i*−1_) given the current token *m*_*i*_ and previous context *c*_*i*−1_ while another function *g* is learned to compute the probability of the next token *m*_*i*+1_, *P*(*m*_*i*+1_|*m*_1_…*m*_*i*_) = *g*(*c*_*i*_) given the current context *c*_*i*_. To improve the modeling performance, we could stack multiple layers of RNNs on top of each other to create a Deep RNN. Each hidden state is continuously passed to both the next time step of the current layer and the current time step of the next layer. The model could still be further improved by using a special type of the hidden layer called Long Short-Term Memory (LSTM) cell to tackle the problem of unstable gradients and handle long sequences. A Deep RNN model with too many hidden layers is quite computationally expensive. Thus, in our experiment, we implemented a model with a stack of 5 hidden states, each hidden state is an LSTM cell with 200 hidden units. We call this model DRnn200-5. We implemented DRnn200-5 using Keras Sequential APIs, TensorFlow 2, and running a Google Colab Pro machine. Note that, the code sequences are used to train both CacheLM and DRnn200-5 are extracted using the Groum model as we described in Section 5.

### 6.2 Recommendation accuracy

In this section, we show the recommendation accuracy of our proposed models and the baselines on top-contributed programmers over 10 selected projects. For each project, we select top-5 contributed programmers by the number of commits. We train and test our models and the baselines for each programmer. Due to a lack of space, we only report top-1 accuracy. Tables 3 and 4 show the top-1 commendation accuracy of top-contributed programmers in the projects intellij-comm and osmand. To report the result in all the selected projects, we compute the average top-1 accuracy of programmers in each project. Table 5 shows the average results in all 10 projects.

**Table 3:** Recommendation accuracy of top-contributed programmers in intellij-comm.

Name	#Commits	PerCR	ProCR	GenCR	PersonaSum	PersonaMax	CacheLM	DRnn200-5
Dmitry Jemerov	15,962	58.5%	55.7%	41.7%	64.3%	63.6%	47.0%	57.5%
Peter	14,615	55.9%	53.2%	39.2%	66.0%	62.9%	48.0%	58.3%
Alexey Kudravtsev	13,934	59.3%	55.3%	40.1%	64.9%	63.0%	47.1%	57.9%
Anna Kozlova	12,589	57.5%	55.8%	39.8%	66.0%	61.4%	46.3%	58.4%
Vladimir Krivosheev	11,784	55.7%	57.0%	40.8%	66.6%	62.5%	47.0%	58.8%

**Table 4:** Recommendation accuracy of top-contributed programmers in osmand.

Name	#Commits	PerCR	ProCR	GenCR	PersonaSum	PersonaMax	CacheLM	DRnn200-5
Victor Shcherb	6,730	53.8%	49.6%	39.1%	63.1%	61.4%	48.2%	55.4%
Weblate	4,029	50.4%	48.8%	38.3%	61.8%	58.4%	47.0%	55.3%
Sonora	3,586	51.9%	47.8%	37.9%	60.5%	60.9%	47.2%	54.4%
Franco	2,536	52.8%	49.2%	34.3%	59.4%	60.8%	45.3%	53.2%
Jan Madsen	2,207	50.1%	46.8%	38.5%	61.7%	62.6%	46.4%	56.6%

**Table 5:** Recommendation accuracy of top-contributed programmers in selected projects.

Project	PerCR	ProCR	GenCR	PersonaSum	PersonaMax	CacheLM	DRnn200-5
liferay-portal	53.7%	51.5%	37.5%	65.3%	63.4%	46.9%	57.9%
intellij-comm	57.4%	55.4%	40.3%	65.6%	62.7%	47.1%	58.7%
osmand	51.8%	48.0%	37.6%	61.3%	60.8%	46.8%	54.3%
geogebra	52.6%	48.5%	35.5%	61.7%	59.8%	46.7%	54.4%
elasticsearch	46.8%	43.8%	36.9%	58.4%	56.4%	42.0%	54.6%
camel	49.5%	45.6%	38.8%	58.1%	56.8%	43.3%	53.2%
lucene-solr	50.2%	49.1%	40.9%	60.5%	58.3%	42.5%	54.9%
cdt	48.3%	44.9%	37.9%	57.2%	55.5%	44.1%	51.8%
cassandra	49.5%	45.5%	37.6%	56.1%	55.2%	43.5%	52.4%
hadoop	50.5%	46.7%	40.9%	60.6%	58.5%	45.7%	54.7%

From the tables, we can see several interesting results. Overall, the personalized model PerCR outperforms the project-level recommendation model ProCR, and the general model GenCR. It generates a 2-3% gap over ProCR and 12-15% gap over GenCR. When incorporating sub-models together, the recommendation accuracy increases significantly with top-1 accuracy approaching 60-65%. The combining method using weighting coefficients (PersonaSum) yields a slightly higher result compared to using the max function (PersonaMax) but the difference is insignificant.

About the baselines, the top-1 accuracy of DRnn200-5 is significantly higher than CacheLM with a gap of around 10%. This shows that DRnn200-5 is a much better approach for modeling sequences. The top-1 accuracy of DRnn200-5 is also better when compared with each sub-model. However, when combining the sub-models, PersonaSum still has higher top-1 accuracy than DRnn200-5 by an average of 4-6%. Overall, the result shows that by combining three simple sub-models that capture personal, project-specific, and common code patterns together, Persona still outperforms the baselines which mostly focus on the common code patterns.

### 6.3 Recommendation accuracy over time

In this section, we evaluate the recommendation accuracy of Persona and sub-models over time. We design the experiment as follows. For a programmer *d*, we divide his set of commits into equal time intervals. A time interval of *t*_*i*_ contains all the commits of the programmer during that time. Depending on the code history of programmers, we could divide the commits into months, quarters, or years. At time interval *t*_*i*_, we will use all the commits of the programmer before *t*_*i*_ to train PerCR, and all the commit of other programmers before *t*_*i*_ to train ProCR. In other words, Persona is trained on all the commits of the project before *t*_*i*_. All the commits in *t*_*i*_ will be used for testing. With this experiment, we want to replicate the real-world accuracy of code recommendation models.

**Fig 5:**
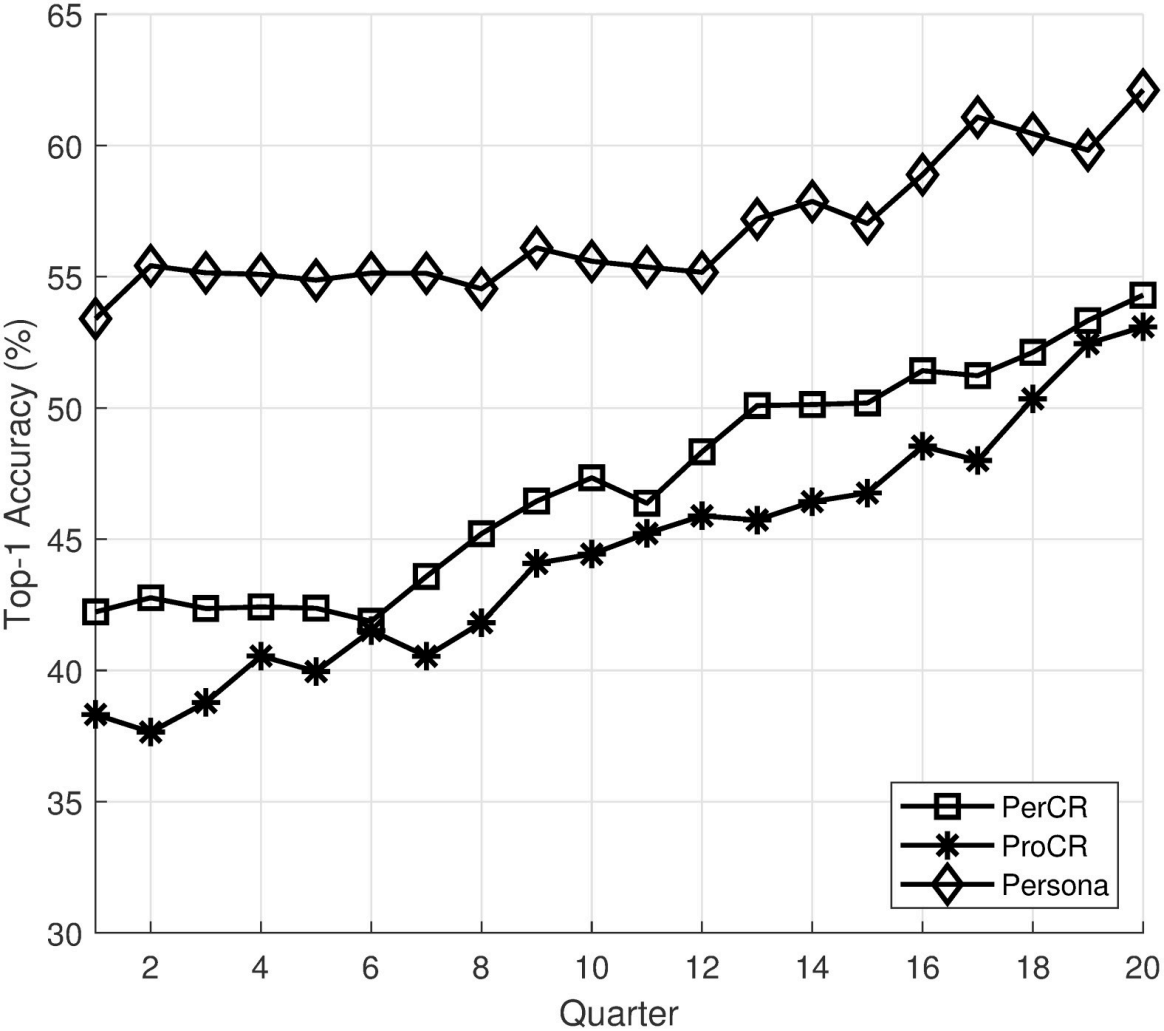
Top-1 accuracy over time of “Dmitry Jemerov”.

**Fig 6:**
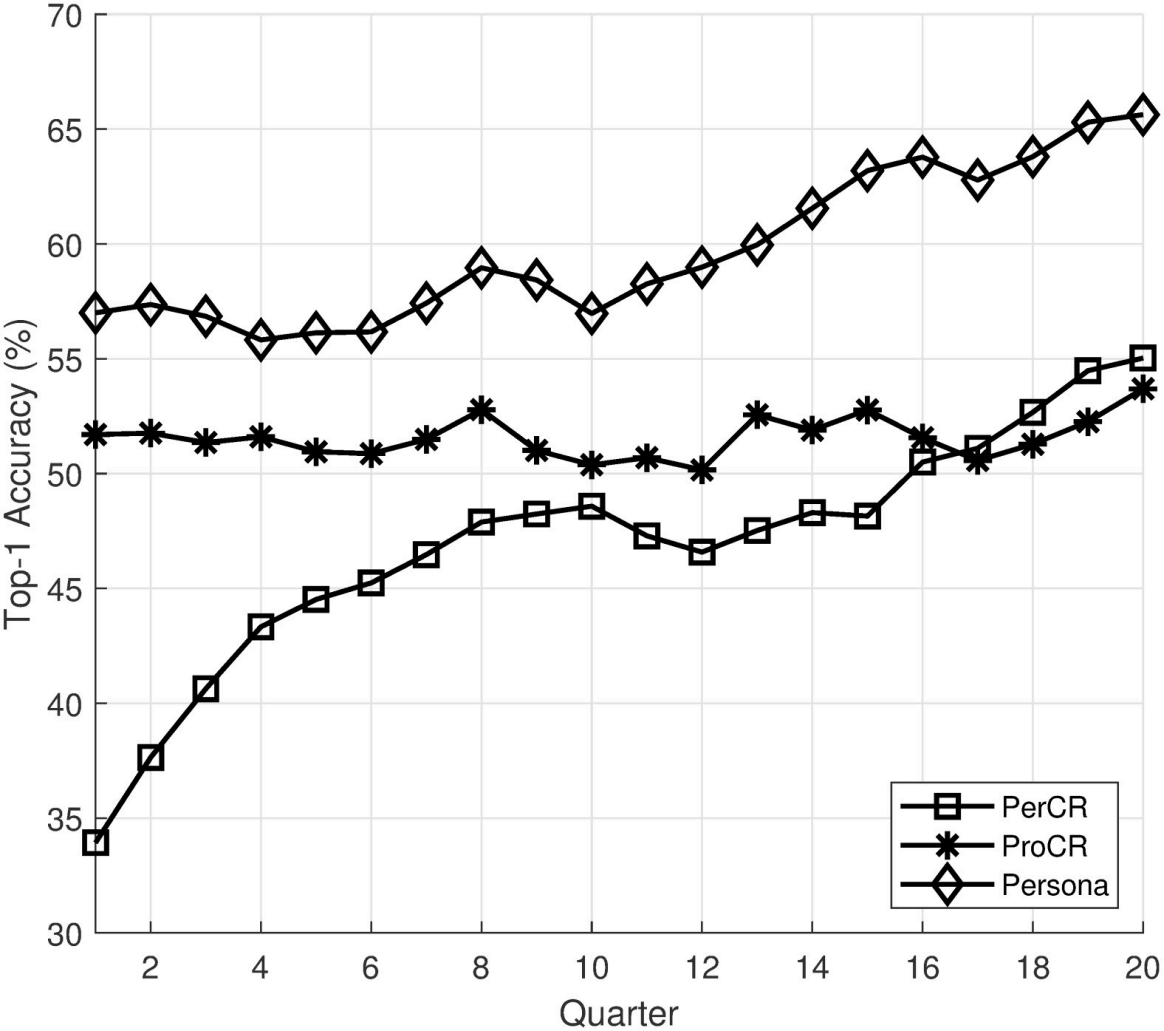
Top-1 accuracy over time of “Vladimir Krivosheev”.

**Fig 7:**
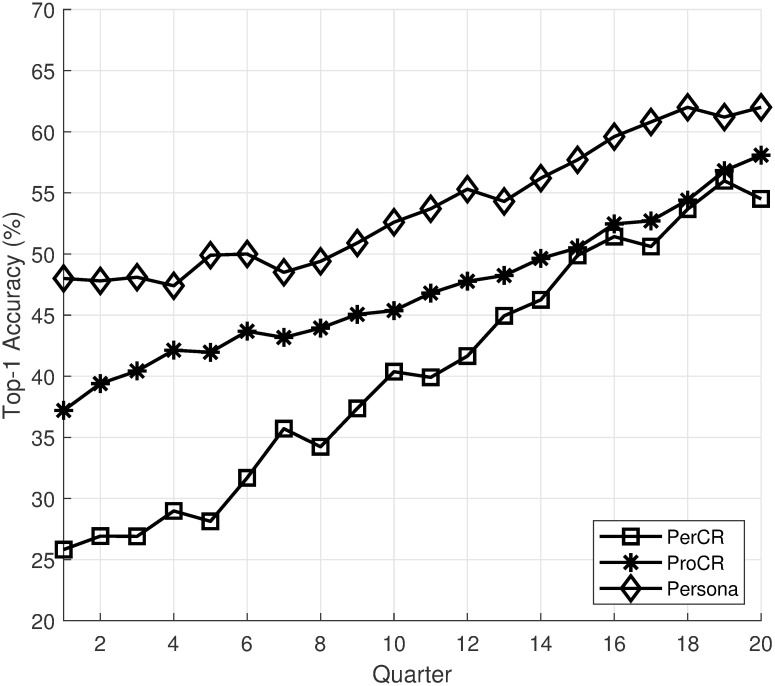
Top-1 accuracy over time of “Alexey Kudravtsev”.

We choose intellij-comm as the subject system. The project has 279,093 commits with 510 contributors spanning from 2005 to now. From the project, we select three programmers with different types of contributions for evaluation. Due to the lack of presentation space, we will use quarters as the time interval, and we only show the recommendation accuracy for the first 20 intervals. Figs 5–7 show the top-1 recommendation accuracy over time for three programmers in the project. For better visualization, we only shows the top-1 accuracy of GenCR, ProCR, and Persona in the figures. The top-1 accuracy of GenCR and the baselines is similar to values reported in Table 3.

From the figures, we can see that the recommendation accuracy of PerCR and ProCR increase over time as these models have more training data. This leads to an increase in the accuracy of Persona. Another interesting observation is that the amount of training data affects PerCR and ProCR significantly. The first programmer (Fig 5) is the main contributor to the project from the start. As he committed a lot of code, his personalized model outperforms the project-level model. The second programmer (Fig 6) joined the project when it already contains most of the code. Thus, his project-level recommendation mode outperforms the personalized model in the beginning. Finally, the third programmer (Fig 7) has a limited contribution at the beginning of the project. His personalized model has low accuracy at the beginning due to a lack of training data. Overall, the experiment shows that the recommendation accuracy of Persona improves over time as more training data is available.

### 6.4 Accuracy on lower-contributed programmers

We have studied the recommendation accuracy of our models on top-contributed programmers. In this section, we study how our models perform when recommending code for lower-contributed programmers. Of course, we do not want to select programmers that committed too little code, as we want to ensure that we have enough training and testing data for the personalized model. Thus, we select the programmers to evaluate as follows. First, for each project, we filter out all programmers with less than 20 commits. Next, we sort programmers by the number of commits and find the median of the list. We select five programmers that have the number of commits right above the median for the study. We train and test our models and the baselines for each programmer. To report the result in all the projects, we compute the average top-1 accuracy of the five programmers in each project.

Table 6 shows the average results in all 10 projects. We could see that the top-1 accuracy of the personalized model PerCR is low due to the lack of training data. The project-level model ProCR still performs reasonably well when compared to other models. This could be explained that the lower-contributed programmers often join the project later when the project has been developed extensively, and they might reuse the project-specific code. Thus, the accuracy of the recommendation model is maintained. On average, Persona still achieves the highest top-1 accuracy when compared to the baselines. For example, PersonaSum has higher top-1 accuracy than DRnn200-5 in 8 out of 10 selected projects with a gap of around 2-5%, while DRnn200-5 just slightly outperforms in the remaining 2 projects. Overall, by incorporating three sub-models together, the Persona performs reasonably well even if the programmers have low contribution in the project or did not join the project for a long time.

**Table 6:** Recommendation accuracy of lower-contributed programmers in selected projects.

Project	PerCR	ProCR	GenCR	PersonaSum	PersonaMax	CacheLM	DRnn200-5
liferay-portal	26.5%	48.7%	37.5%	58.6%	56.8%	46.2%	51.3%
intellij-comm	24.2%	51.4%	39.8%	57.3%	57.1%	49.8%	54.4%
osmand	22.7%	45.3%	35.7%	57.0%	52.8%	47.2%	53.4%
geogebra	20.7%	44.8%	35.5%	56.3%	54.1%	46.0%	53.7%
elasticsearch	15.3%	41.4%	36.9%	47.2%	49.7%	41.1%	47.8%
camel	20.1%	43.6%	38.8%	52.4%	47.9%	42.9%	49.6%
lucene-solr	17.5%	46.5%	40.9%	53.9%	52.5%	42.0%	51.3%
cdt	21.7%	42.0%	37.9%	48.3%	48.7%	43.5%	49.2%
cassandra	15.6%	42.9%	37.6%	51.0%	49.6%	42.6%	49.3%
hadoop	16.5%	43.1%	40.9%	55.6%	52.4%	44.9%	51.4%

### 6.5 Ablation study

In this section, we perform an ablation study to understand the contribution of the sub-models to the recommendation performance of Persona. In particular, we focus on the model PersonaSum. Similar to the first experiment, we measure the recommendation accuracy of PersonaSum with different configurations on top-contributed programmers over 10 selected projects. For each project, we select top-5 contributed programmers by the number of commits, then we compute the average top-1 accuracy of programmers in each project. As described in Section 4, in PersonaSum, we combine sub-models using Eq 5 where *α*_1_ + *α*_2_ + *α*_3_ = 1 are weighting coefficients. The values of *α*_1_, *α*_2_, *α*_3_ represent the contribution level of PerCR, ProCR, and GenCR correspondingly. Removing a sub-model from the system equals to setting *α*_*i*_ = 0. For example, if we remove ProCR from the system, *α*_2_ is set to 0, which means $\alpha_{1}=\alpha_{3}=\frac{1}{2}$. The model is called PerCR+GenCR. Similarly, if we remove ProCR and GenCR from the system, *α*_2_ and *α*_3_ are set to 0, which means *α*_2_ = 1. The model become PerCR.

Table 7 shows the average top-1 recommendation accuracy when removing one or two sub-models from the system. Note that, as we use the same settings as the previous experiment, the results for PerCR, ProCR, GenCR, PersonaSum are the same as in Table 5. We have several interesting observations. Firstly, if we remove two sub-models from the system, the sub-model PerCR outperforms the project-level recommendation model ProCR, and the general model GenCR. Secondly, if we remove a sub-model from the system, PerCR+GenCR has the highest top-1 accuracy. PerCR+GenCR also has a significantly higher top-1 accuracy when compared to then each sub-model, especially, GenCR. This result shows that although GenCR has low top-1 accuracy, combining this sub-model with others could improve the recommendation significantly. Finally, we could see that PerCR+ProCR does not have much improvement when compared to each sub-model.

**Table 7:** Recommendation accuracy by removing one or two sub-models from Persona.

Project	PerCR	ProCR	GenCR	PerCR+ProCR	ProCR+GenCR	PerCR+GenCR	PersonaSum
liferay-portal	53.7%	51.5%	37.5%	56.6%	55.8%	61.1%	65.3%
intellij-comm	57.4%	55.4%	40.3%	59.1%	58.3%	60.7%	65.6%
osmand	51.8%	48.0%	37.6%	54.3%	50.2%	55.9%	61.3%
geogebra	52.6%	48.5%	35.5%	53.9%	52.1%	56.8%	61.7%
elasticsearch	46.8%	43.8%	36.9%	48.9%	47.3%	53.8%	58.4%
camel	49.5%	45.6%	38.8%	51.1%	48.5%	53.4%	58.1%
lucene-solr	50.2%	49.1%	40.9%	52.6%	51.9%	57.4%	60.5%
cdt	48.3%	44.9%	37.9%	50.6%	48.6%	54.0%	57.2%
cassandra	49.5%	45.5%	37.6%	49.9%	49.2%	53.8%	56.1%
hadoop	50.5%	46.7%	40.9%	51.5%	50.8%	56.1%	60.6%

### 6.6 Weighting coefficients

**Fig 8:**
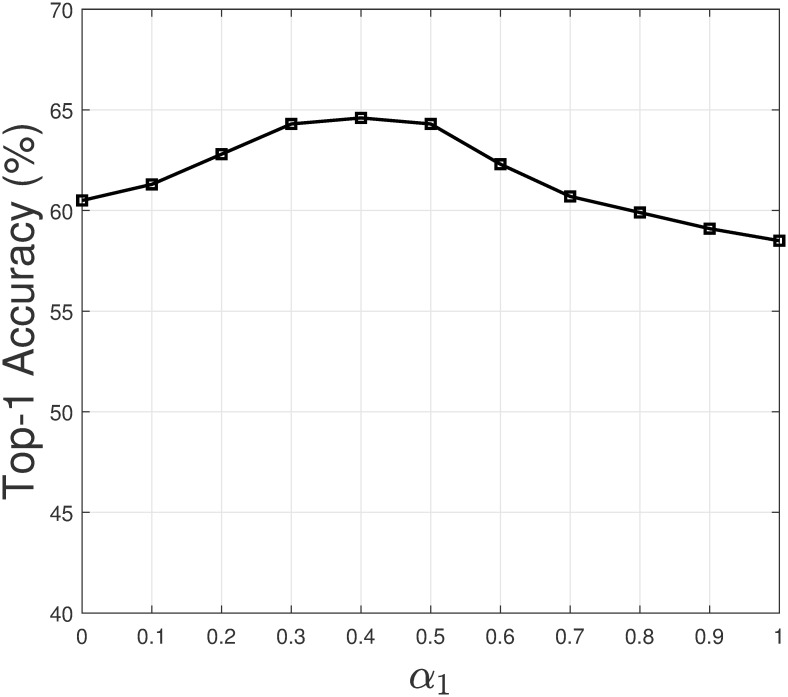
Top-1 accuracy when *α*_1_ changes.

**Fig 9:**
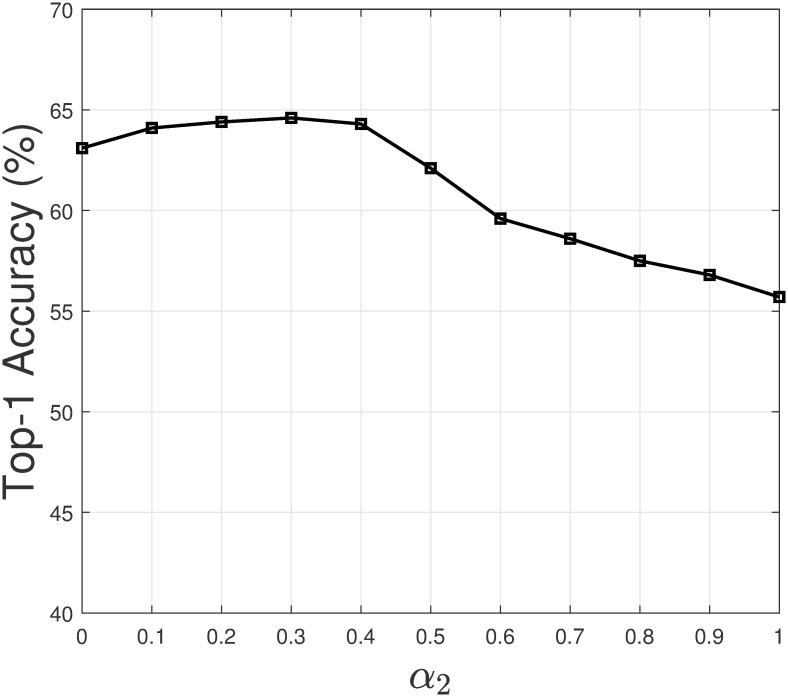
Top-1 accuracy when *α*_2_ changes.

**Fig 10:**
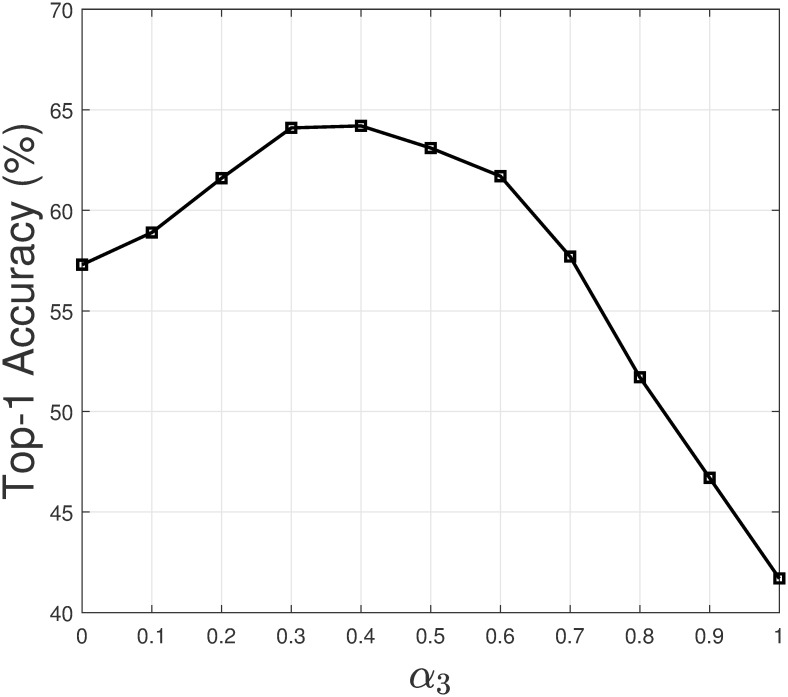
Top-1 accuracy when *α*_3_ changes.

In this section, we study how the values of weighting coefficients affect the recommendation result of the model. As a personalized model, Persona is built and updated for each programmer. We selected the top-1 programmer by the number of commits in the intellij-comm project to study the weighting coefficients. Let us assume we choose the weighting coefficient *α*_1_ to study. Note that the weighting coefficients have a constraint *α*_1_ + *α*_2_ + *α*_3_ = 1. For each value of *α*_1_, we set $\alpha_{2}=\alpha_{3}=\frac{1-\alpha_{1}}{2}$. Next, we let *α*_1_ takes different values from 0 to 1, and increase by 0.1. Then we evaluate the top-1 accuracy of PersonaSum based at each value of *α*_1_. Fig 8 shows how top-1 accuracy changes when *α*_1_ changes. A similar process is repeated for the remaining weighting coefficients. Figs 9 and 10 show the result for *α*_2_ and *α*_3_.

From the figures, we have several observations. Firstly, if a weighting coefficient has a high value (closer to 1), the top-1 accuracy tends to decrease. In such a case, the top-1 accuracy of PersonaSum is dominant by a sub-model. Secondly, the top-1 accuracy result is most sensitive with the value of *α*_3_. When *α*_3_ is high, the result decreases significantly as the weighting of GenCR increases in PersonaSum. Additionally, we could see that the result is often high if all the weighting coefficients are in the range [0.3, 0.5]. These observations are valuable in selecting the values of weighting coefficients to improve the recommendation accuracy of Persona.

## 7 Discussion

In this section, we discuss several aspects of Persona in more detail. From the machine learning perspective, Persona is a simple ensemble approach with three sub-models: PerCR, a model that captures personal code patterns of a programmer; ProCR, a model that captures the project-level code patterns that the programmer is working on; and GenCR, a general model that capture code patterns shared between multiple projects. Persona learns and recommends like *n*-gram and RNN but more flexibly. For example, fuzzy membership functions are not probability distribution functions, thus, Persona does not need to normalize ∑_*e*_ *μ*_*f*_(*c*) = 1.

We could consider Persona is a fuzzy logic system specially designed for the software engineering domain. It represents code patterns as fuzzy logic rules. It uses fuzzy set theory to model and apply those rules, and uses fuzzy union operations to combine the rules. In the traditional fuzzy logic system, variables are often continuous such as Temperature, Density or linguistic like LOW, VERY LOW. The membership functions are often manually defined by domain experts with functions such as triangular or trapezoidal. In Persona, variables are discrete, i.e. class, method, etc., and the membership functions are estimated automatically.

In our evaluation, we re-implemented (CacheLM) as the baseline method. Although we tried to replicate the same settings as the previous research [4, 15], the recommendation results of the baseline models in our evaluation are different when compared to the original research. The dissimilarity could be explained due to the differences in several factors including the dataset, cross-validation, recommendation tasks, etc. Similarly, in our implementation of DRnn200-5, we used different configurations with the previous studies [4, 16] so the result is not comparable.

The evaluation suggests that Persona outperforms the baseline models such as DRnn200-5 which reaffirms our earlier assumption. As a crowd-based approach, DRnn200-5 infers and recommends common code patterns from a large code corpus while ignoring the difference in coding preferences of programmers. When such differences are blurred, the performance of the recommendation tool for a specific programmer is hurt. Persona achieves high accuracy because it takes into consideration the personal coding preferences of programmers while also captures the project-specific and common code patterns. In our future work, we plan to incorporate personal coding patterns with the model such as DRnn200-5 to further improve the recommendation accuracy.

In Persona, we combine the sub-models using both Eqs 5 and 6. In the first method, we set the weighting coefficients equally, $\alpha_{1}=\alpha_{2}=\alpha_{3}=\frac{1}{3}$. These weighting coefficients determine the contribution of a sub-model to Persona. We performed a study on how values of weighting coefficients affect the recommendation result of the model, which reveals several insights. Different combinations of weighting coefficients could be experienced to optimize the performance of the model. In our future work, we plan to develop a method to estimate such optimal coefficients.

## 8 Conclusion

To help programmers work more productive, modern IDEs often include code recommendation features. There are multiple techniques have been proposed to further improve the effectiveness of current recommendation tools. Most of the methods focus on modeling and extracting common code patterns that often appear in a large pool of available source code. However, each programmer has certain coding preferences and styles. These preferences are personal and might differ between programmers. Such personal preferences should be taken into consideration when providing code recommendations as it could improve the effectiveness and enhance user satisfaction. We propose Persona, a novel personalized code recommendation model while also combines with project-specific and common code patterns. As a personalized model, Persona is built and updated for each programmer. It utilizes the fuzzy set theory to model correlation/association between code elements. The empirical evaluation suggests that our recommendation tool based on Persona is highly effective. It outperforms the baseline models in the task of recommending the next identifier in a code sequence.
